# Non-operative management of early onset small urethrocutaneous fistula post hypospadias repair: a systematic review

**DOI:** 10.1007/s00345-026-06504-z

**Published:** 2026-06-17

**Authors:** Benhard Christopher Simanjuntak, Putu Angga Risky Raharja, Gerhard Reinaldi Situmorang, Irfan Wahyudi, Arry Rodjani, Omar S. A. J. H. Aljarallah, Muthana Al-Salihi, Santiago Andres Vallasciani, Tariq O. Abbas

**Affiliations:** 1 Department of Urology, Faculty of Medicine, Cipto Mangunkusumo Hospital, Universitas Indonesia, Jakarta, Indonesia; 2 Division of Pediatric Urology, Department of Surgery, Sidra Medicine, Doha, Qatar; 3 Weill Cornell Medical College, Doha, Qatar; 4 College of Medicine, Qatar University, Doha, Qatar

**Keywords:** Non-operative management, Urethrocutaneous fistula, Hypospadias repair, Tissue adhesive, Fibrin glue, Recatheterization

## Abstract

**Background:**

Urethrocutaneous fistulas (UCF) after hypospadias repair are typically managed surgically, but conservative approaches may offer a less invasive, cost-effective alternative. While surgical repair remains the standard, non-operative strategies may eliminate anesthesia exposure, shorten recovery time, and lower healthcare costs. However, systematic evidence regarding these approaches remains limited.

**Methods:**

This narrative systematic review evaluates non-operative management for small (typically ≤ 2 mm) UCFs. A comprehensive search was conducted across PubMed, Cochrane, ScienceDirect, Wiley, and Google Scholar up to April 2025. The study followed PRISMA guidelines and is registered in PROSPERO (CRD420251069870). We included randomized controlled trials (RCTs), comparative trials, case series, and anecdotal reports involving children (< 18 years). Risk of bias was assessed using Cochrane RoB-2, ROBINS-I, and JBI Critical Appraisal tools.

**Results:**

Five studies involving 73 patients were included for analysis. Due to significant clinical heterogeneity in fistula characteristics and interventions, a pooled closure rate was not calculated. Reported individual study closure rates ranged from 53.8% to 62.5% in the primary series, with one anecdotal report (*n* = 1) showing 100% success. Success was more frequently observed in early-onset UCFs (diagnosed within 14 days post-catheter removal) and those with a diameter ≤ 2 mm. Minor adverse events included localized heat (42.8%) and transient dysuria (4.7%). Cost-analysis from one RCT indicated that non-operative adhesive application cost approximately 9.3% compared to total operative costs. Most included studies were classified as having a high risk of bias.

**Conclusion:**

Non-operative approaches using tissue adhesives, fibrin glue, or recatheterization may be viable for small (≤ 2 mm), early-onset UCFs. However, the current evidence is limited, heterogeneous, and carries a high risk of bias. These findings should be considered hypothesis-generating, and further high-quality comparative studies are required to establish definitive clinical protocols.

**Trial registration:**

PROSPERO CRD420251069870.

## Background

Hypospadias is one of the most frequent congenital anomalies of the urogenital system, occurring in approximately one in every 200 to 300 male live births [1]. Various surgical techniques and decision-making algorithms have been proposed to guide urethroplasty procedures [2]. However, complications following hypospadias repair are common. Among these, urethrocutaneous fistulas (UCF) and meatal stenosis are the most frequently observed complications, even in cases handled by experienced surgeons [3].

One of the most challenging complications following hypospadias repair is UCF, with incidence rates ranging from 0% to over 35%. On average, UCF occurs in approximately 7.5% of cases, though this percentage varies depending on the surgeon’s expertise and the surgical technique employed [4]. A 2024 meta-analysis by Ismail et al. involving 2,040 patients found that UCF was the most common complication, occurring in 14.07% of cases (287 patients; range: 11.00%–18.02%). Their analysis showed high heterogeneity (I² = 75.72%, 95% CI: 59.95%–85.28%; *p* < 0.0001) [5]. Several risk factors include meatal stenosis, urethral stricture, inadequate waterproofing, improper choice of sutures, needles, and suturing techniques, tension at the suture line, poor tissue handling, and infection [6].

UCF is typically managed through surgical interventions. Most literature emphasizes surgical techniques and their outcomes. For instance, studies discuss various surgical methods such as simple closure, layered closure, and the use of waterproofing layers to repair UCF [7]. While surgical repair remains standard, non-operative strategies may eliminate anesthesia exposure, shorten recovery time, and lower costs. However, there is limited information available on non-operative approaches for managing UCF post-hypospadias repair.

Over the years, several non-surgical approaches have been developed for treating UCF. Various cyanoacrylate-based adhesives have been explored, including isobutyl, isohexyl, and 2-octyl cyanoacrylate (OCA). These adhesives possess tensile strength along with bacteriostatic and hemostatic properties. Among them, N-butyl-2-cyanoacrylate (NBCA) and OCA are the most used, with OCA exhibiting tensile strength comparable to sutures by day seven. In urology, NBCA has been utilized for arteriovenous fistula embolization, segmental renal embolization, vas deferens occlusion in sterilization, urinary fistula management, and as a suture alternative in circumcision [8]. Additionally, early urethral recatheterization has been proposed to facilitate spontaneous UCF closure by reducing tension and promoting healing before epithelialization occurs [9]. Despite these emerging options, high-quality evidence supporting conservative treatment remains limited. Therefore, this review aims to evaluate the available evidence on non-operative management strategies for small, early-onset UCFs after hypospadias repair.

## Methods

This systematic review is in accordance with PRISMA guidelines (Preferred Reporting Items for Systematic Reviews and Meta-Analyses) and *Cochrane handbook for systematic reviews of interventions* [10]. The objective of this manuscript is to review the non-operative managements of UCF after hypospadias repair. The review is registered to PROSPERO with ID number CRD420251069870.

### Search strategy

We searched the following sources for relevant literature published since the inception of each database. The keywords used were (“Hypospadias/surgery“[MeSH] OR hypospadias[tiab]) AND (“Urethrocutaneous fistula“[tiab] OR “urethral fistula“[tiab] OR “cutaneous fistula“[tiab] OR “fistula“[MeSH]). The last search date for all databases was 1 April 2025. The literature search was conducted using several reputable databases to ensure comprehensive coverage. These included PubMed, covering publications from the late 1940s to the present; ScienceDirect (Elsevier), with content dating from 1947 to the present; the Cochrane Controlled Trials Register, specifically the 4th Edition published in April 2019; Wiley Online Library, encompassing literature from 1807 to the present; and Google Scholar, which has been indexed since 2004.

If we discovered new keywords during the literature search, we adjusted our electronic search strategy to incorporate them and recorded the modifications. Additionally, we explored reference lists from included trials, reviews, and meta-analyses to identify other potential studies or relevant publications. Duplicates from various databases were swiftly eliminated, and then the titles and abstracts were screened. Subsequently, the full texts of the remaining studies were evaluated based on the pre-established eligibility criteria.

### Eligibility criteria

This study is conducted as a narrative systematic review to evaluate non-operative management for urethrocutaneous fistulae (UCF). We included randomized controlled trials (RCTs), comparative trials, and case series. To address the scarcity of evidence in this field, we included five studies involving 73 patients. The population of interest consists of children (< 18 years) with UCF diagnosed within approximately 14 days post-catheter removal (‘early-onset’). While our primary focus remained on fistulae ≤ 2 mm, we included data from Lapointe et al. by extracting only the subgroup of patients meeting this size criterion. Additionally, the case report by Wen-zeng et al. (*n* = 1) was retained to provide a comprehensive landscape of available literature but is explicitly labeled as anecdotal evidence.

The review also gives priority to early-onset UCF, which is defined as a fistula found within approximately 14 days after catheter removal. This timing is physiologically important, because the fistula tract in this early phase is usually composed of active granulation tissue rather than mature epithelium and is therefore more amenable to non-operative measures. For the last synthesis, studies had to have a minimum follow-up duration of 6 months after intervention to ensure the stability of the closure and because of the well-acknowledged risk of late failure. No restrictions were imposed on the publication status or language of the studies. All non-English literature was translated into English by a professional translation service to ensure the accuracy of the translation.

### Data extraction

Two review authors independently screened abstracts and titles using Covidence software to identify studies requiring further evaluation. They assessed all potentially relevant records, including full texts, mapped records to studies, and categorized them as included, excluded, awaiting classification, or ongoing, following the criteria outlined in the Cochrane Handbook for Systematic Reviews of Interventions [11]. Any disagreements were resolved through consensus or by consulting a third review author. If no resolution was reached, the study was marked as ‘awaiting classification’. Data from each study recorded includes authors’ names, publication year, sample size, patient type, study design, closure rates, recurrence rates, complication rates, and cost-effectiveness analysis.

### Quality of studies and bias assessment

Qualitative analysis and reporting of data from the studies were performed in accordance with the guidelines contained in the Cochrane Handbook for Systematic Reviews of Interventions [11]. To assess the risk of bias, randomized trials were assessed with the revised tool for Risk of Bias in randomized trials (RoB-2) for RCTs, ROBINS-I for non-randomized comparative studies, and the JBI Critical Appraisal Checklist for case series to ensure appropriate quality evaluation across different study designs [12, 13]. Risk of bias was independently assessed by two reviewers (AP and BN). Discrepancies were resolved by discussion, and if consensus could not be reached, a third reviewer (TA) adjudicated.

Due to the significant clinical heterogeneity observed in fistula characteristics and the varied non-operative interventions across the included studies, a quantitative meta-analysis was deemed inappropriate. Therefore, this study is explicitly conducted as a narrative systematic review, focusing on a qualitative synthesis of the outcomes to provide a pragmatic clinical overview.

### Evidence hierarchy and weighting

Evidence was weighted according to clinical hierarchy to account for the inherent variability in quality of studies. The randomised controlled trial (RCT) was considered as the highest level of evidence and was regarded as the primary source, while clinical trials and case series were considered as supporting evidence. Data from the single-patient trial (*n* = 1) were also classified and labelled only as “anecdotal evidence” so as to not artificially inflate the overall effectiveness rates.

### Outcome

Primary outcomes include fistula closure (success) rate and recurrence rate. Secondary outcomes comprise complication rates and cost-effectiveness. Closure and recurrence rates were observed for each study at minimum 6 months postoperatively. For studies reporting interim results at 3 months, the latest available follow-up data (e.g., 12 months or yearly) were prioritized for the final success rate analysis. Complications included procedural or medical complications after UCF management and were assessed using the Clavien-Dindo classification. Cost-effectiveness was assessed using appropriate cost of non-operative management tailored to each study. Owing to clinical heterogeneity across study designs and interventions, a pooled closure rate was not calculated. Closure rates are instead reported descriptively per study with 95% confidence intervals, in keeping with the narrative-review framework of this study (Table 6).

### Data analysis

Meta analysis could not be performed due to varying methods of intervention, unclear definitions of terminology in our included studies, such as early timing and fistulae sizes, and reported outcomes. Due to the identified conceptual heterogeneity, results were stratified into subgroups based on timing of onset and fistula size. This stratification allowed for a more robust evaluation of success rates across different clinical scenarios.

## Results

### Study selection

**Fig. 1:**
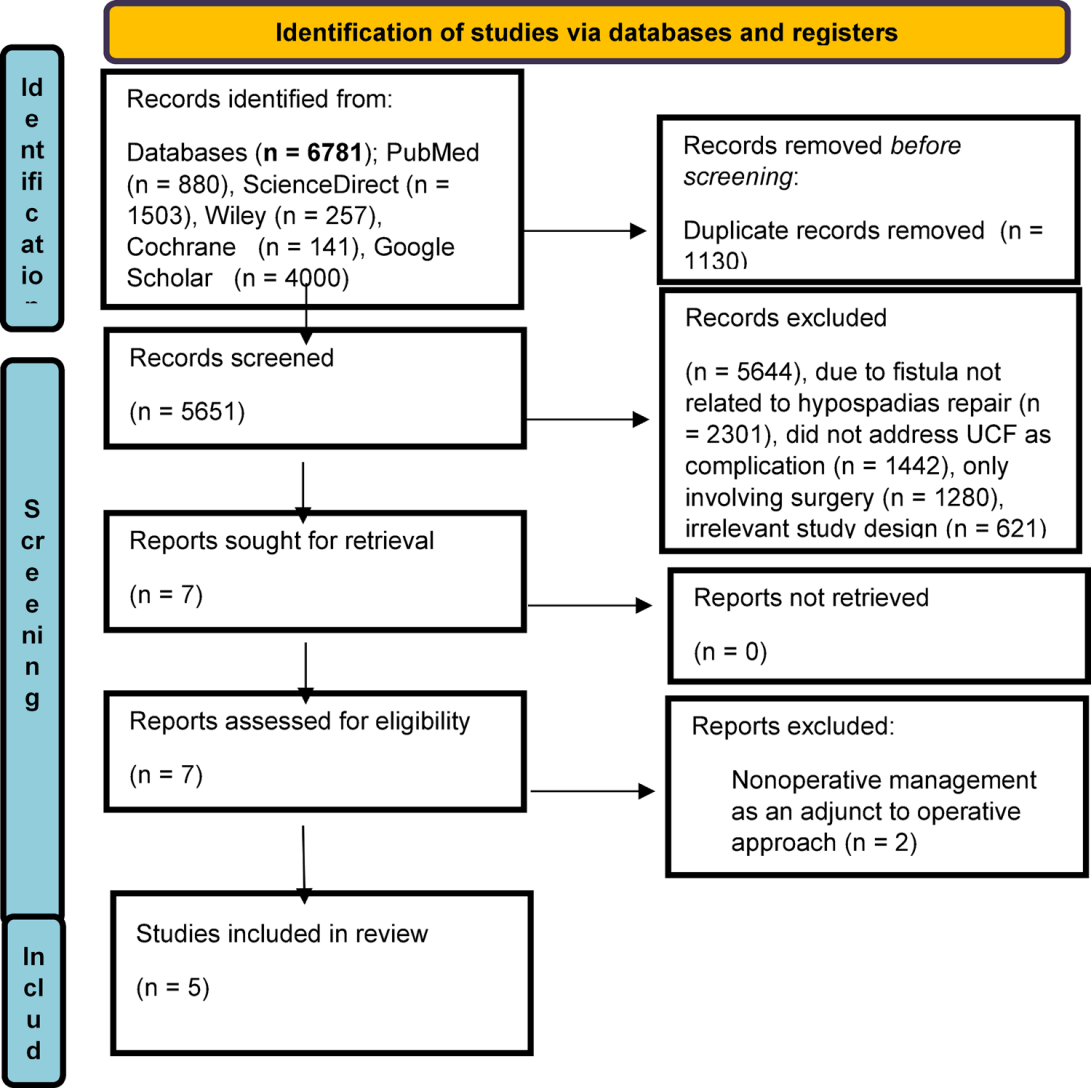
Literature search algorithm

The literature search initially identified a total of 6,781 references, incorporating results from electronic databases as well as additional references obtained through manual searches and trial registries. Following the removal of 1,130 duplicate records, a total of 5,651 references were subjected to title and abstract screening. During this process, studies that did not meet the predefined eligibility criteria were excluded. After careful evaluation, 7 references were potentially relevant and reviewed to the full-text screening. Two references, Gopal et al. and Higazy et al. were excluded because both the usage of fibrin glue in hypospadias repair and cyanoacrylate in UCF repair, respectively, were used intraoperatively and henceforth not in accordance with our pre-defined eligibility. Ultimately, 5 studies met the eligibility criteria and were incorporated into the qualitative synthesis. The process of study selection is summarized in the PRISMA flow diagram (Fig. 1).

### Study characteristics

We identified 1 randomized controlled-trials (RCTs) (Ambriz-Gonzalez et al.), 3 clinical trials (Chandrasekharam et al., Lapointe et al., Wen-zeng et al.), and 1 case series (Prestipino et al.) [8, 9, 15–17]. For details, please refer to the ’Characteristics of included studies’ table, Table 1. The included trials reported the use of non-operative management of UCF post hypospadias repair, with or without comparative intervention. We contacted the corresponding authors of these studies to get any additional information and received responses (Table 2).

**Table 1:** Summary of findings for Outcomes for Long-standing UCF (≤ 2 mm)

Study; Design	Group	Characteristics of patients	Follow-up	Initial location of hypospadias		Initial hypospadias repair	Characteristics of fistulas	Details of intervention		Reported outcome		
Ambriz-Gonzalez et al. (2014); RCT	OCA vs. surgery	Number: 21 vs. 21 Age: 28.6 ± 24.5 mo vs. 35.4 ± 17.6 mo Long-standing UCF (at least 6 months post-surgery)	12 mo vs. 12 mo	9 (43%) proximal, 7 (33%) medial, 5 (24%) distal vs. 10 (48%) proximal, 6 (29%) medial, 5 (24%) distal		3 Duckett, 5 MAGPI, 3 Onlay, 9 Snodgrass, 1 Thiersch-Duplay vs. 3 Duckett, 3 MAGPI, 1 Onlay, 9 Snodgrass, 5 Thiersch-Duplay	Number: 1.3 (1–4) Diameter: 2.96 ± 1.0 mm Vs. Number: 1.1 (1–2) Diameter: 3.82 ± 0.89 mm	Triamcinolone cream to reduce inflammation and edema. Outpatient treatment after midazolam sedation (0.5 mg/kg orally) and Foley catheterization (10 Fr, balloon inflated with 1.5 ml of water). Gentle scarification of the fistula edges was made with a 27-g needle and cotton swabs to produce slight bleeding. OCA adhesive was applied using forceps, followed by multiple thin layers. If persistence was noted after 5 days, OCA application was repeated.		60% vs. 68% closure rates 42.8% local heat, 4.7% dysuria vs. NA 14,809 vs. 158,538 USD		
Prestipino et al. (2011); Case series	NBCA (Late Subgroup)	Number: 7 Age: 42.6 mo	1.5-5 years	1 midshaft; 6 subcoronal		TIP urethroplasty	Diameter: ≤ 2 mm in 2 cases; > 2 mm in 5 cases	Topical anesthetic, scarification of edges with 27G needle, followed by multilayer NBCA application.		53.8% (7/13) closure rates		

**Table 2:** Summary of findings for Outcomes for Early-onset UCF (≤ 2 mm)

Study; Design	Group	Characteristics of patients	Follow-up	Initial location of hypospadias	Initial hypospadias repair	Characteristics of fistulas	Details of intervention	Reported outcome
Chandra-sekharam et al. (2016); Trial	Re-catheterization	Number: 9 Age: 1–9 years Early UCF ( 2 weeks post initial surgery)	1 mo, 3 mo, and yearly	NA	5 Duplay, 4 Onlay island flap	Location: 4 coronal, 3 midpenile, 2 penoscrotal	Children underwent urethral calibration and catheter reinsertion. They received antibiotics and oral anticholinergics. The catheter was removed after two weeks.	66% closure rates
Lapointe et al. (2002); Case series	Spontaneous closure	Number: 12 Age: 10 mo − 12 years	3–6 months	NA	TIP and others	Diameter: ≤ 2 mm (subgroup extracted)	Observation only for spontaneous closure after early identification of fistula.	58.3% (7/12) closure rates
Wen-zeng et al. (2011); Anecdotal	Anecdotal (*n* = 1)	Number: 1 Age: 3 years	6 months	Mid-penile	TIP urethroplasty	Diameter: 1 mm (single fistula)	Conservative management with prophylactic antibiotics and local hygiene.	100% (1/1) closure rate
Prestipino et al. (2011); Case series	NBCA (Early Subgroup)	Number: 6 Age: 22.6 mo	5–7 years	2 subcoronal; 4 anterior shafts	TIP urethroplasty	Diameter: ≤ 2 mm in 5 cases; > 2 mm in 1 case	NBCA applied directly without sedation/anesthesia after disinfecting the fistulae.	66.7% (4/6) closure rates

### Risk of bias in included studies

**Fig. 2:**
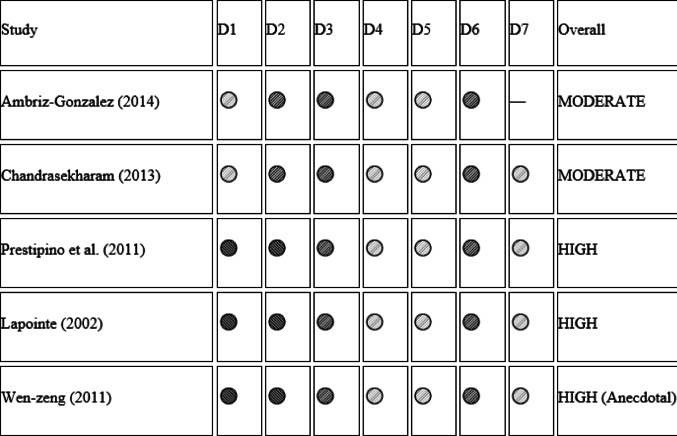
Risk of Bias Summary (Traffic Light Plot)

Risk of bias was assessed using tools tailored to each study design to ensure methodological transparency. The RCT by Ambriz-Gonzalez (2014) showed a moderate risk of bias via the Cochrane tool. However, the assessment for non-comparative studies and case series was updated to reflect a higher risk profile (Fig. 2). Non-RCT studies evaluated with ROBINS-I were categorized as having a high risk of bias, particularly regarding confounding and selection of participants. Specifically, the study by Wen-zeng (2011) is flagged as having a high risk of bias due to its nature as anecdotal evidence (*n* = 1). Similarly, while the JBI Checklist was applied to the case series by Prestipino et al. (2011) and Lapointe et al. (2002), these were ultimately classified as having a high risk of bias to acknowledge the inherent limitations of non-comparative data and the potential for selection bias in patient recruitment (Tables 3, 4, 5).

**Table 3:** Assessment of risks of bias of RCT

Studies	Random sequence generation	Allocation concealment	Blinding of participants and personnel	Incomplete outcome data	Selective reporting	Other sources of bias	Overall risk of bias
Ambriz Gonzalez, 2014	Moderate	Low	Low	Moderate	Moderate	Low	Moderate

**Table 4:** Assessment of Risks of Bias of Comparative and Observational Studies (ROBINS-I)

Studies	Domains							Overall
	Con-founding bias	Selection bias	Measure-ment bias	Bias due to deviation from intended intervention	Bias due to missing data	Bias in measure-ment of outcome	Bias in selection of the reported result	
Chandrasekharam, 2013	Moderate	Low	Low	Moderate	Moderate	Low	Moderate	Moderate
Lapointe, 2002	High	High	Low	Moderate	Moderate	Low	Moderate	High
Wen-zeng, 2011	Critical	Critical	Low	Moderate	Moderate	Low	Moderate	High (Anecdotal)

**Table 5:** Assessment of risks of bias of comparative case series studies

No	Question (JBI Critical Appraisal Item)	Assessment: Prestipino et al. (2011)
1	Were there clear criteria for inclusion in the case series?	**Yes**: Patients with early or long-standing UCF post-TIP urethroplasty were specified.
2	Was the condition measured in a standard, reliable way for all participants?	**Yes**: Identification was consistently based on clinical urinary leakage from the fistula.
3	Were valid methods used for identification of the condition for all cases?	**Yes**: Clinical diagnosis and direct observation of the fistula site were used for all cases.
4	Did the case series have consecutive inclusion of participants?	**Unclear**: The study does not explicitly state if recruitment was consecutive over the study period.
5	Did the case series have complete inclusion of participants?	**Yes**: All 13 identified patients were included in the intervention and follow-up.
6	Was there a clear reporting of the demographics of the participants?	**Yes**: Specific age groups (mean 22.6 and 42.6 months) were reported.
7	Was there a clear reporting of clinical information of the participants?	**Yes**: Initial hypospadias repair (TIP) and specific fistula locations were detailed.
8	Were the outcomes or follow-up results clearly reported?	**Yes**: Success rates and follow-up (1.5–7 years) were clearly defined for both subgroups.
9	Was there a clear reporting of the presenting site(s)/clinic(s) demographic information?	**Yes**: Information was provided as a single-center experience.
10	Was statistical analysis appropriate?	**N/A**: The study primarily utilized descriptive percentages for closure rates.

Overall Appraisal: Moderate-High (Risk of Bias: High)

### Characteristics of subjects

The included studies are characterized by a limited number of samples in their respective intervention groups, which necessitates a cautious interpretation of the reported success rates. The randomized controlled trial by Ambriz-Gonzalez et al. featured the largest intervention cohort with 21 samples treated with OCA. In the observational and comparative studies, the sample sizes were notably smaller: Prestipino et al. included 13 patients treated with NBCA, and Chandrasekharam et al. reported on 9 patients who underwent conservative management via recatheterization.

The evidence base is further supplemented by smaller series and anecdotal reports: Lapointe et al. contributed data for 8 patients (specifically focusing on the ≤ 2 mm subgroup for this review), while the report by Wen-zeng et al. provided anecdotal evidence from a single patient (*n* = 1) treated with topical fibrin glue. Collectively, while these 73 cases provide a foundational landscape for non-operative management, the paucity of large-scale, multi-center data remains a significant limitation in establishing a definitive clinical protocol.

### Initial location and repair of hypospadias

Only two studies, Ambriz-Gonzalez et al., and Prestipino et al., reported location of initial hypospadias. Both have different location of hypospadias, but Ambriz-Gonzalez had higher rate of closure rates, 60% vs. 42.8% in long-standing UCF. Approach of hypospadias repair mostly used conventional techniques, except for Wen-zeng et al. which utilized tissue patch stage I urethroplasty and Prestipino et al. with TIP urethroplasty.

### Characteristics of fistula

The four studies reporting diameter had fistula size less than 5 mm. Prestipino et al. used 2 mm as a cut-off categorization, as other studies have also utilized. Whether smaller (≤ 2 mm) UCF is an appropriate cut-off for the decision to perform nonoperative management and whether it may result in higher closure rates in unclear. It should be noted that authors may also have selection bias to recruit patients with less than 5 mm UCF in leaning toward nonoperative approach.

### Closure and recurrence rates

Closure rates, reported by all five studies, range from 53.8% to 100%. The lowest closure rates of 53.8% were reported by Prestipino et al. in three patients with long-standing UCF treated with NBCA observed within 1.5-5 years of follow-up. The highest closure rates are 62.5% in a study by Lapointe et al., where 6 patients were treated with NBCA [15]. Recurrence rate within follow-up period were not reported in any of the included studies. In all studies, patients with persistent UCF were treated surgically after 6–12 months, in accordance with local protocol.

### Complication rates

In an RCT by Ambriz-Gonzalez et al., nine (42.8%) patients reported sensation of local temperature increase while 1 (4.7%) reported 2 days of transient dysuria. Wen-zeng et al. reported no complication (local urinary leakage, red swelling, hot pain) within 6 months postoperatively. Other studies did not report complication rates, limiting the ability to draw definitive conclusion regarding complication rates of non-operative approach [8,9,15,17].

### Cost-effectiveness analysis

Only Ambriz-Gonzalez et al. reported cost-effectiveness analysis, comparing the total costs of adhesive applications were 14,809 USD and total costs of reoperations to achieve successful UCF closure were 158,538 USD over 21 patients. This calculation shows that adhesive application costs approximately 9,3% compared to total operative costs. No other authors reported cost-effectiveness analysis in their studies.

### Follow-up period

The follow-up period was not standardized. Chandrasekharam et al. follow-up method was 1 month, 3 months, and yearly thereafter of history and clinical examination. Wen-zeng et al., Lapointe et al., Ambriz-Gonzalez et al. follow-up period was for 2–6 months, 6 months, and 12 months, respectively. Follow-up period for Prestipino et al. was 5–7 years for early fistula and 1.5-5 years for late-standing fistula.

### Quantitative synthesis of success rates

Table 6 provides a descriptive per-study summary of closure rates with 95% confidence intervals. Owing to clinical heterogeneity in fistula characteristics, study designs, and interventions, no pooled estimate was calculated. Reported closure rates ranged from 53.8% in the mixed early-and-late cohort of Prestipino et al. to 62.5% in the early-onset cohort of Chandrasekharam et al., with an additional anecdotal single-patient report (Wen-zeng et al.) describing successful closure. Subgroup observations support a more favourable trend in early-onset, ≤ 2 mm fistulae compared with long-standing or larger lesions. However, given the limited evidence base (five studies, 73 patients) and the high risk of bias in most non-comparative studies, these per-study rates should be interpreted descriptively as hypothesis-generating rather than as a basis for clinical decision-making.

**Table 6:** Descriptive Summary of Closure Rates by Study (95% CI)

Study (Year)	Clinical Subgroup	Success/Total	Proportion (%)	95% Confidence Interval*
Ambriz-Gonzalez (2014)	Long-standing	9/15	60.0%	[32.3, 83.7]
Chandrasekharam (2013)	Early	20/32	62.5%	[43.7, 78.9]
Prestipino et al. (2011)	Mixed (Early/Late)	7/13	53.8%	[25.1, 80.8]
Lapointe et al. (2002)	Early (≤ 2 mm subgroup)	5/8	62.5%	[27.7, 84.8]
Wen-zeng et al. (2011)	Anecdotal (*n* = 1)	1/1	100.0%	[2.5, 100.0]

## Discussion

Various cyanoacrylate-based adhesives, such as isobutyl, isohexyl, and OCA offer tensile strength along with bacteriostatic and hemostatic properties. While both NBCA and OCA are widely used, OCA demonstrates superior tensile strength, comparable to sutures after seven days [18–20]. In Prestipino et al., the adhesive remained in place for 4 to 7 days, with no variation between early and long-standing UCF. The tensile strength of cyanoacrylate kept the fistula edges tightly approximated. Cyanoacrylate maintained edge contact, promoting healing, while NBCA created a waterproof barrier against urine, supporting granulation [17]. While NBCA application yielded good results, preventing UCF formation remains the ideal outcome.

Application of cyanoacrylates tissue adhesive are practical and ease of use, which can be done painlessly in an outpatient setting. The discomfort caused by catheterization was avoided by administering 0.5 mg/kg of oral midazolam 45 min prior to the procedure. To reduce edema before applying the cyanoacrylate, authors used triamcinolone, which has been shown to reduce inflammation. Although corticosteroids can potentially hinder the healing process, their use was short-term, and no complications in wound healing were observed [15].

Temporary re-catheterization can aid spontaneous UCF closure by allowing the neourethral suture line to rest, keeping the fistula dry, reducing edema and inflammation, and promoting wound healing. Wen-zeng et al. recommended the catheter to be kept for at least 7 days after operation, while a longer duration may prevent the occurrence of postoperative urethral stricture [16]. Daher et al. found that a 3-week catheterization after hypospadias repair led to fewer complications than a 1-week duration. Studies also show urethral healing is a slow process, continuing for up to 21 days [21]. Extending catheterization in some cases may improve neourethral healing [9].

Conservative management is also an option. Bloesch et al. reviewed the traditional linear closure and purse-string suture of UCF following hypospadias repair over a 24-year period. In their study of operative managements, he found that 6/62 patients (9.6%) had resolution of UCF in conservative manners, 3 spontaneously and 3 after a single dilation. In distal or mid-penile hypospadias, spontaneous resolution occurred in 3 patients at 5, 7, and 23 months after hypospadias repair. They reported that if the fistula is very small (only 1–2 mm) and there is no distal obstruction, it is advisable not to immediately close it, as spontaneous closure is possible — one fistula closed on its own 23 months after hypospadias repair [22].

Our review indicates that non-operative managements are best suited for small (preferably ≤ 2 mm) UCF. In Prestipino et al., fistulas ≤ 2 mm were more successfully repaired than those > 2 mm (five of seven vs. two of six). The treatment was more effective in early (four of six) than in long-standing UCF (three of seven). The fistula location did not appear to impact success. Additionally, fistulas were more likely to respond well to initial application[17].

Based on our included studies, there are several suggestions for when to utilize non operative UCF management. We suggest that the use of non-operative management techniques is given to those with early UCF, defined as 2–3 days after catheter removal or approximately 9–14 days after primary hypospadias repair. The fistula should be less than 5 mm in diameter, either regular or irregular shape. The location of fistula maybe irrelevant, as our studies have demonstrated that non operative management was able to treat various location of fistula (coronal, subcoronal, anterior shaft, midshaft, penoscrotal). The patient selection criteria were those younger than 5 years old, have undergone hypospadias repair surgery or penis extension, with postoperative urinary leakage, and/or were not candidate for the second repair surgery because of a small amount of local tissues.

Timing on when to choose the non-operative approach is early UCF. Prestipino et al. reported that application of NBCA may be better on early fistula due to having initial granulation tissue, while edges of long-standing fistula need to be scarified to initiate a new granulation process [17]. Lapointe et al. utilized similar timing, with application of NBCA on fistulas that were developed 1 to 3 days after stent removal [15]. Wen-zeng et al. found fistula in a patient after application of urinary catheter for 7 days after hypospadias repair [16]. Chandrasekharam et al. also utilized re-catheterization in children with fistula within 2 weeks of initial catheter removal [9].

Fistula site seems irrelevant for the success of non-operative approach. Regarding the initial hypospadias, only 2 studies mentioned the initial location of the hypospadias, 4 mentioned the fistula size, and 3 report the location of fistula. Position of the original meatus in Ambrez-Gonzalez et al. were 9 (43%) proximal, 7 (33%) medial, 5 (24%) distal vs. 10 (48%) proximal, 6 (29%) medial, 5 (24%) distal.^8^ The position of meatus in Prestipino et al. were 2 subcoronal and 4 anterior shafts in early fistula and 1 midshaft and 6 subcoronal in long-standing fistulae [17]. Lapointe et al. has proposed that cyanoacrylate closure could achieve greater success in fistulas located on the penile shaft compared to those at the coronal region, as the shaft skin provides more favorable dermal support and vascularization. The firmer dermal structures may enhance approximation of the tract margins and facilitate secondary epithelialization while the edges are securely maintained in position by the cyanoacrylate [15].

Fistula sizes are relevant for further studies and have not been fully explored in these studies. Small fistula (≤ 2 mm in diameter) were more easily repaired than larger fistula (> 2 mm in diameter) [17] Lapointe et al. did not recommend closure with cyanoacrylate larger than 5 mm [15]. This may be explained by the mechanism of cyanoacrylate to close fistula, as edges of the fistula are held tight due to their capability of tensile strength. might close spontaneously.

Adhesive products may offer a potential alternative for UCF treatment in selected patients, allowing outpatient management without sedation or anesthesia. They cause minimal pain, discomfort, and psychological stress compared to surgical intervention, which requires hospitalization and carries inherent risks. Additionally, failed adhesive treatment does not prevent future surgical procedures if needed. The economic benefits are significant, as adhesives provide a cost-effective, repeatable option without surgical risks. Re-catheterization may further support spontaneous healing of early UCFs, potentially eliminating the need for additional surgery [8,15,17].

Non-operative approach has several contraindications. Fistulas located near the coronal margin may be relatively contraindicated for NBCA treatment and could be more effectively managed after a six-month healing period followed by a repeat glansplasty. Successful closure of a fistula requires adequate skin vascularization, so previous failed surgical attempts may also serve as a relative contraindication for using cyanoacrylate. While no clear pattern was found between fistula size and closure success, we recommend avoiding cyanoacrylate for fistulae > 2 mm. Larger thresholds (e.g., 5 mm) reported in earlier studies are noted historically but were not adopted in this review[.^17^.]

Overall, the risk of bias was moderate for the single RCT (Ambriz-Gonzalez et al.) and high for all non-comparative studies (Chandrasekharam et al., Lapointe et al., Prestipino et al., and the anecdotal report by Wen-zeng et al.). The RCT had unclear random-sequence generation, incomplete outcome data, and selective reporting of complication rates in the control group. The non-comparative studies were limited by the absence of demographic adjustment, lack of independent supervision, incomplete data, a high potential for selection bias inherent to non-comparative designs, thereby heightening departures from intended interventions. Some studies also have incomplete data, thereby prone to bias due to missing data and reporting bias. Due to the inherent limitations of small-scale case series and the potential for selection bias, the overall level of evidence was downgraded. Most non-comparative studies were judged to have a high risk of bias, necessitating a cautious interpretation of the clinical outcomes.

We also noted that there are many heterogeneities in the included studies, namely procedural steps of non-operative management, reported outcome, and follow-up duration and standardization, in each study makes analysis to be difficult. This highlights the need for uniform and clear defining methods and outcome for future studies. We suggested OCA and NBCA application to follow the protocol from Ambriz-Gonzalez et al. Re-catheterization should follow steps from Chandrasekharam et al. Overall complication should be classified with Clavien-Dindo system to ease subgroup analysis. Cost-benefit analysis should be done regarding the costs of utilized non-operative management, i.e. OCA, NBCA, fibrin glue, and catheterization costs, vs. re-operative management for UCF closure in their respective institute. Follow-up duration should be standardized for at least 6 months and preferably 12 months.

Novelty is our study’s strength as this is an early explorative effort to review the non-operative managements of UCF after hypospadias repair. Nonetheless, the study has several limitations. First, it is difficult to draw a concise recommendation on the usage of non-operative approach to manage UCF, as there are varying methods in our included studies. Second, the small number of available studies (only 5 out of 5651 initial searches) made our conclusion hard to validate, suggesting the need for further research with similar method to be performed first. Third, there are low number of patients (sample size) in some study, especially Wen-zeng et al. (2011) which only had 1 patient as a trial. Therefore, further studies aiming for meta-analysis should aim to elucidate closure rates by weighting studies by their sample size. Fourth, while unlikely, it is possible that additional studies have been conducted but remain unpublished, or that we may have missed existing studies despite our thorough search efforts. The lack of standardized long-term follow-up across all studies remains a limitation. Although late failure is a recognized risk in UCF repair, the high success rates observed at 6–12 months in this review provide a preliminary basis for non-operative strategies in selected cases. Future research should mandate a 12-month follow-up to definitively confirm spontaneous closure stability.

## Conclusion

Surgical repair remains the gold standard for urethrocutaneous fistulae following hypospadias repair. The available evidence reviewed here suggests that non-operative approaches — including tissue adhesives (OCA, NBCA), fibrin glue, and recatheterization — may serve as a potential alternative for carefully selected patients with early-onset (≤ 14 days post-catheter removal) and small (≤ 2 mm) UCF. However, the current evidence base is limited to five studies (*n* = 73), is clinically heterogeneous, and most non-comparative studies carry a high risk of bias. Reported closure rates ranged from 53.8% to 62.5% across primary series, with one anecdotal single-patient report showing 100% success. These findings should be regarded as hypothesis-generating rather than as a basis for definitive clinical protocols. Further high-quality, prospective comparative trials with standardized definitions of fistula size and timing, and a minimum 6-month (preferably 12-month) follow-up, are warranted to confirm the role of non-operative management in selected paediatric patients.

## Data Availability

All data generated or analyzed during this study are included in this published article and its supplementary materials. The review protocol was registered in PROSPERO (CRD420251069870).

## Abbreviations

CI: Confidence interval
Fr: French (catheter size)
I²: I-squared statistic (measure of heterogeneity)
MAGPI: Meatal advancement and glanuloplasty incorporated
MeSH: Medical subject headings
mo: Months
NA: Not available
NBCA: N-butyl-2-cyanoacrylate
OCA: 2-octyl cyanoacrylate
PRISMA: Preferred reporting items for systematic reviews and meta-analyses
PROSPERO: International Prospective Register of Systematic Reviews
RCT: Randomized controlled trial
RoB-2: Revised tool for risk of bias in randomized trials
ROBINS-I: Risk of bias in non-randomized studies of interventions
TIP: Tubularized incised plate
UCF: Urethrocutaneous fistula
USD: United States Dollar
